# Lipoprotein-associated phospholipase A2 (Lp-PLA2) – possible diagnostic and risk biomarker in chronic ischaemic heart disease

**DOI:** 10.1080/14756366.2020.1839447

**Published:** 2020-11-13

**Authors:** Adriana Diaconu, Bogdan-Ioan Coculescu, Gheorghe Manole, Horațiu Vultur, Elena Claudia Coculescu, Cristina Maria Stocheci, Ioan-Sorin Tudorache, Alexandra-Ligia Dincă, Valeriu Gabi Dincă

**Affiliations:** a Faculty of Medicine, Carol Davila University of Medicine and Pharmacy, Bucharest, Romania; b Faculty of Medicine, Titu Maiorescu University, Bucharest, Romania; c Center for Military Medical Scientific Research, Bucharest, Romania; d Colentina Clinical Hospital, Bucharest, Romania; e Faculty of Dental Medicine, Carol Davila University of Medicine and Pharmacy, Bucharest, Romania; f Faculty of Sciences, University of Pitesti, Pitesti, Romania; g Medicover Hospital, Bucharest, Romania

**Keywords:** A2 phospholipase (PLA2); arachidonic acid (AA); chronic ischaemic heart disease; oxidative stress, biomarker

## Abstract

In a group of 208 patients with chronic ischaemic heart disease, the variation of A_2_-associated-LDL phosphatase (Lp-PLA_2_) serum concentration values was analysed in dynamics at a two-week interval. The conclusion of the study is that the values of serum concentration of Lp-PLA_2_ can be accepted as a biomarker with diagnostic specificity for chronic ischaemic heart disease, a parameter of real utility in medical practice, both in situations where the patient, although clinically reporting the existence of angina pectoris, does not show specific changes on an EKG, and for the assessment of the response to personalised therapy.

## Introduction

Globally, the goals of cardiovascular disease research after the start of the current millennium are to identify simple or complex disease-specific markers that can be used to predict risk in atherosclerosis^1^. Regardless of the clinical form of the disease under which it is expressed, research in atherosclerosis with coronary localisation is focussed on the study of parameters and processes that provide data on the relationship with: inflammation, intracellular signalling, cardiovascular remodelling, apoptosis and similar cardiomyocyte death processes^1–4^. At the level of the hypoxic myocardium, the development of such processes under the action of locally developed oxidative stress presupposes as a first stage the alteration of the existing fluidity at the level of the proteo-phospholipid layers in the membrane constitution^5^. The process involves, as a first stage, the peroxidation of membrane ceramides generating hydroperoxides which, acting on membrane proteins, modify their physical properties, including the orientation and dynamics of constitutional phospholipids. An essential contributor is the flow of phospholipids from the deep layers of the membrane to the outer membrane, a flow subsequently compensated by a balancing on both sides^6^.

**Figure 1.**
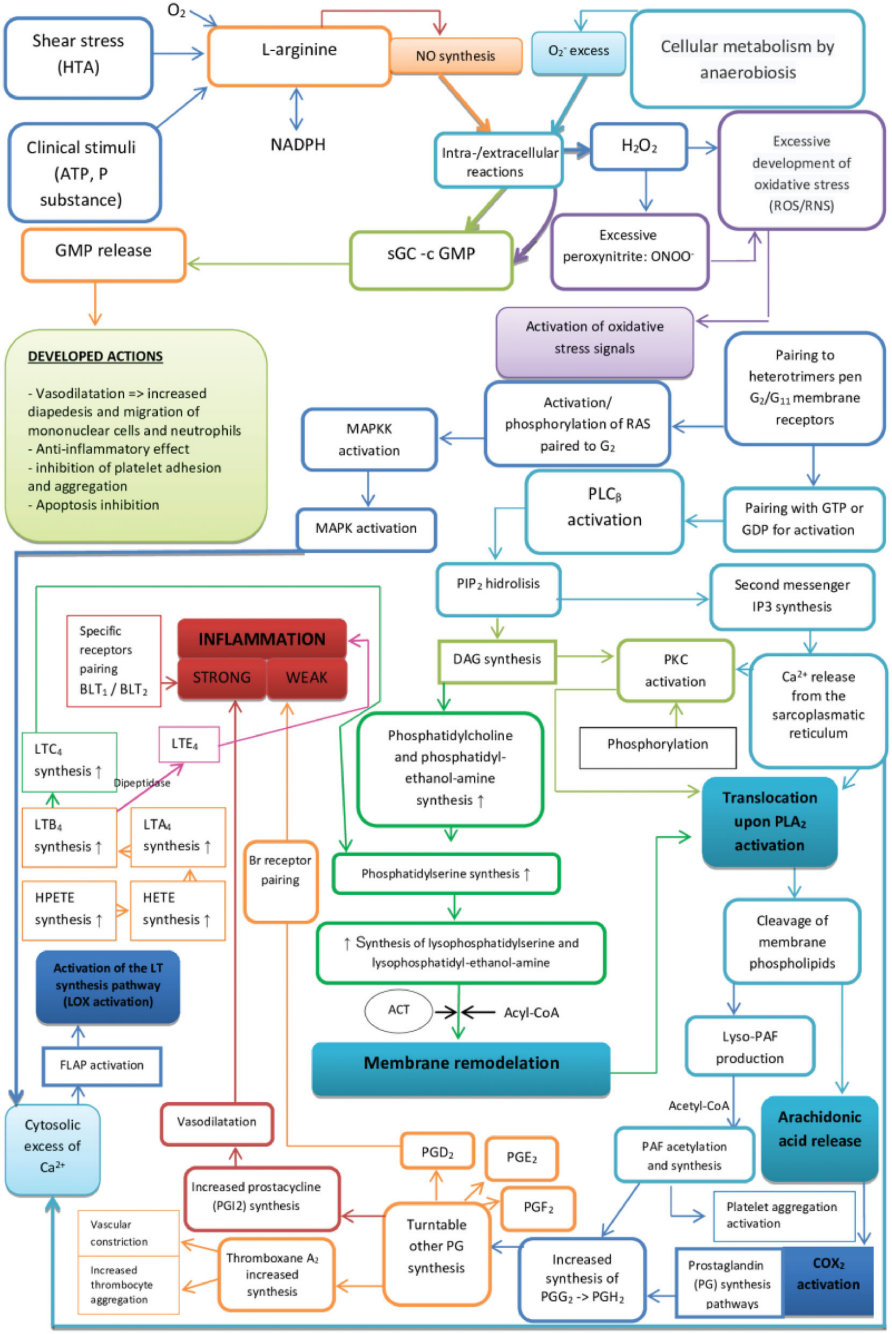
The involvement of PLA_2_ in the process of peroxidation of membrane lipids; the synthesis of the main mediators initiating inflammation in atherogenesis. FLAP: 5-lipoxygenase-activating protein; 5-HPETE: 5-hydroperoxyeicosatetraenoic acids; HETE: hydroxyeicosatetraenoic acids; cPLA_2_: cytosolic phospholipases A_2_ cytosolic; PLC: phospholipase C; PIP_2_: phosphatidylinositol 4,5-bisphosphate; IP_3_: phosphatidylinositol; DAG: diacylglycerol; PKC: proteinkinase C; ACT: acyltransferases; PG: prostaglandins; LT: leukotrienes; lyso-PAF: alkyl homolog PAF; PAF: platelet-activating factor (receptor); LOX: lipoxygenases; MAPK: MAP-kinases (MEK, ERK), mitogen-activated protein kinase; c GMP: Guanosine monophosphate 3′,5′-cyclic.

The structural-functional integrity of cells is dependent on the maintenance of redox homeostasis, as redox systems develop modulation effects on the signalling cascade at the cellular level^7–9^. In the conditions of redox homoeostasis disorder, regardless of the type of cells, the way they respond depends on the intensity of the oxidative stress, but also on its anti-oxidant defence capacity. To maintain redox homeostasis, in case of excessive increase in intracellular ROS/RNS concentration, modulatory mechanisms that develop antioxidant activity or reduce the processes induced by oxidative stress are used. Their efficiency expresses the degree of stimulation of the genes that induce the synthesis of enzymes recognised as developing such actions^10^^,^^11^. Similarly to what occurs in any cell exposed to oxidative stress and cardiomyocytes, the development of processes is mediated by the activation of serine-lipase enzymes located in their membranes or cytosol, but also secreted by some migrated cells at the level of myocardium, such as: monocytes/macrophages, T lymphocytes and mast cells^12–14^. At their level, oxidative stress causes the activation of PLA_2_ and C-type enzymes in the structure of the membranes or the cytosol of the myocardial fibre. The PLA_2_ superfamily comprises of 19 members, of which at least 13 have been identified with certainty. Important are the isozymes belonging to the secretory PLA_2_ (sPLA_2_), cytosolic PLA_2_ (cPLA_2_/PLA_2_ group IV) and calcium-independent PLA_2_ (iPLA_2_) groups^13–16^. It is accepted that approximately 80% of PLA_2_ circulates bound to low-density lipoprotein (LDL) and only 20% binds to high-density lipoprotein (HDL) or other circulating lipoprotein fractions^15^. The role of the lipoprotein component in the sPLA_2_ complex is unknown, but X-ray studies have shown that in this form the enzyme is much more active by binding to the layers of membrane phospholipids or to mycelium^17^. Coupling the substrate to the active site of the membrane-bound enzyme may have different structural-functional consequences, because after the release of the ester bond from the sn-2 position of the oxidised glycerophospholipids and the release of the arachidonic acid, under catalytic action it becomes a precursor of eicosanoids, generating biologically active compounds^14–19^ (Figure 1).

From the previous figure, the processes of translocation and activation of PLA_2_ and the synthesis of prostaglandins (PG), leukotrienes (LTs) and lysophospholipids^20^ are highlighted as “turntables” in the development of atherosclerosis. The resulting compounds activate, sequentially, various intracellular message transduction pathways, down to the nucleus level, developing the initiation and maintenance of the inflammatory process in atherosclerosis, including the one with coronary localisation^21^. This allows us to claim that the Lp-PLA_2_ enzyme, which is pro-atherogenic through its circulating concentrations, provides the medick, indirectly, with information about the amplitude of the atherosclerotic processes.

The effects mentioned are the result of the regional presence of oxidative stress, but also of endothelin (ET) which, when associated, develops a potentiating role. In optimal tissue concentrations, this does not develop action on the normal heart, but when present at high values it develops complex effects, mediated in autocrine or paracrine ways, depending on the subtype of the activated membrane receptors. The action is possible because cardiomyocytes express both types of endothelin receptors: ET_A_ and ET_B_. At the level of the myocardium that works in optimal irrigation conditions (physiological conditions), the first type of receptors is predominant, so that in conditions of tissue hypoxia the ET_B_ subtype is overexpressed, which explains the effects of endothelin delaying the diastolic relaxation of the myocardium, cardiomyocyte hypertrophy, interstitial fibrosis, and induction of certain foetal patterns of gene expression responsible for cytoprotection, apoptosis, or necrosis^22^^,^^23^. The scientific support for claiming such actions are developed by endothelin is represented by the results of determinations that showed in the hypertrophied or hemodynamically overloaded myocardium a rich representation of the endothelin system, because the optimal oxygen requirement is not offered. What is still unknown is whether ET in myocardial excess is produced only by atherosclerotically affected coronary endothelial cells, or if it is also the result of excessive synthesis and secretion at the myocardial fibre level ^22^^,^^24^. An argument in support of this last action is the results of studies that have shown that the plasma level of endothelin increases progressively with the degree of cardiac decompensation. However, the answer to the question of whether endothelin hyper-synthesis occurs as a compensatory mechanism or, on the contrary, is one that precipitates the development of myocardial contractile function deficit remains unclear^22^.

## Material and methods

The study group consisted of 208 patients (147 men and 61 women, respectively) suffering from chronic ischaemic heart disease, excluding cases of acute myocardial infarction or sequelae.

**Figure 2.**
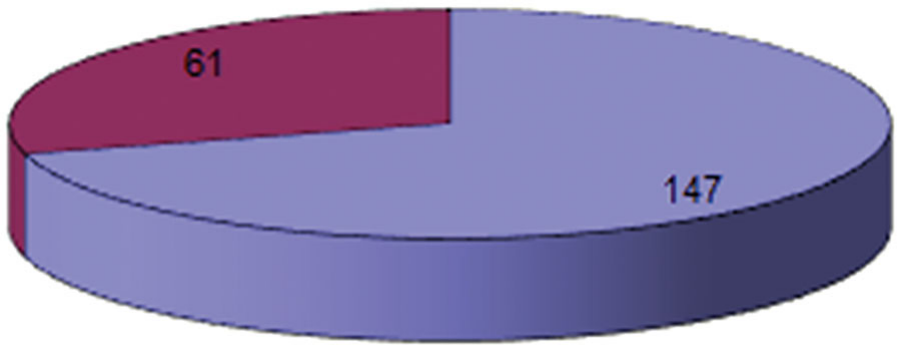
Distribution of cases by sex and age group.

The present study appoints as a prospective one following ethical principles derived from the Declaration of Helsinki (Committee on Human Investigation). The age limits were between 35 and 64 years, the average being 54.3 years (Figure 2).

In selecting the patients co-opted into the group, their weight was taken into account, relating it to the ideal one. The selection followed the instructions of the experts of the National Institute of Health, from the WHO report published in 1989, by calculating the value of body mass index (BMI), based on the calculation formula:

$$\text{BMI}\;={\;\text{Body}\;\text{weight}\;\text{expressed}\;\text{in}\;\text{kg}/\text{height}}^{2}={\;\text{Kg}/m}^{2}.$$

Only patients with chronic ischaemic heart disease falling into the first three weight classes were included in the group, so not the obese.

In order for the patients in the study to have relatively the same diet and lifestyle, the patients were hospitalised for a period of 2 weeks, during which time they received the same diet and identical coronary dilating and lipid-lowering medication. Patients in the study were given exact doses of circulating levels of Lp-PLA_2_.

The dosage of Lp-LPA_2_ serum was determined both at admission and at discharge on the 14th day. Depending on when the determination was made, the following were calculated for the whole batch:

- medium serum concentration on the first day of hospitalisation;
- medium serum concentration on the last day of hospitalisation (the 14^th^ day);

Subsequently, using the two average values that were calculated, a third parameter was admitted - the average serum concentration of Lp-PLA2 per batch, regardless of the timing of the dosage. Mention: The procedure with two determinations instead of one was used in order to obtain a value as true as possible for the medium serum concentration/batch, but also to be able to appreciate the effect of the established therapy on the value of the serum concentrations of the studied parameters.

The principle of the method of determination: Lp-PLA_2_ hydrolyses the ester group at position 2 of the substrate [1-myristoyl-2-(*p*-nitrophenylsuccinyl) phosphatidylcholine], releasing *p*-nitrophenylsuccinate, a compound which in an aqueous solution releases *p*-nitrophenol. The concentration of *p*-nitrophenol released is measured spectrophotometrically and is directly proportional to the activity of the enzyme. Spectrophotometric measurements were performed at 405 nm and 505 nm. The variation of the absorption per minute is used to calculate the enzymatic activity. The results were expressed in IU/L (µmols/min/L).

## Results

a. In the studied group, regardless of sex, the values of the average serum concentration of Lp-LPA_2_ calculated based on the values of the determinations performed at hospitalisation, discharge and the whole group (regardless of the time of determinations) are shown in the following Table (Table 2).

**Table 2.** Calculated medium values of Lp-PLA_2_ serum activity in the study group, regardless of sex.

Name/unit of measurement		Normal biomarker values	Calculated medium value/batch depending on		
			Timing of dosing		Regardless of the timing of dosing
			Hospitalisation	Discharge	
Lp – PLA_2_	IU/L	225.65 ± 20.8	295.34 ± 17.9	286.65 ± 19	290.73 ± 20.67

b. The incidence of the increased medium concentration of Lp-LPA_2_-emy per the whole group, regardless of the time of determination was 163/208 patients, representing a percentage of 78.3 (Figure 3).

**Figure 3.**
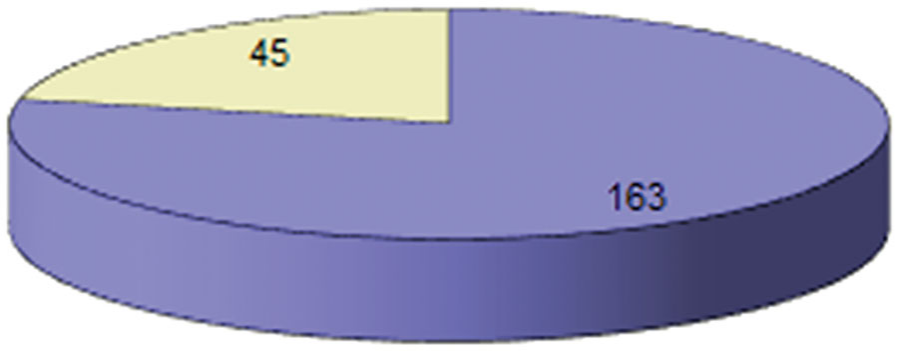
Distribution in the studied group of calculated medium Lp-LPA_2_-emy serum values, regardless of the time of dosing and the sex of the patients. Number of component cases of the batch (n = 208); number of patients with an increased value of the average concentration of Lp-LPA2-emy/whole group, regardless of the time of determination (n = 163); no. of patients with the normal value of the medium concentration of Lp-LPA2-emy/whole group (n = 45).

c. In relation to the sex of the patients, regardless of the time of collection, the incidence of hyper-LPA_2_ is distributed as follows:

- 43 women (70.5% of female cases or 20.67% of the entire study group);
- in men, hyper-Lp-PLA_2_ was present in 120 patients out of 147 (81.7%), the equivalent of 55.6% of the case series of the whole group (Figure 4).

**Figure 4.**
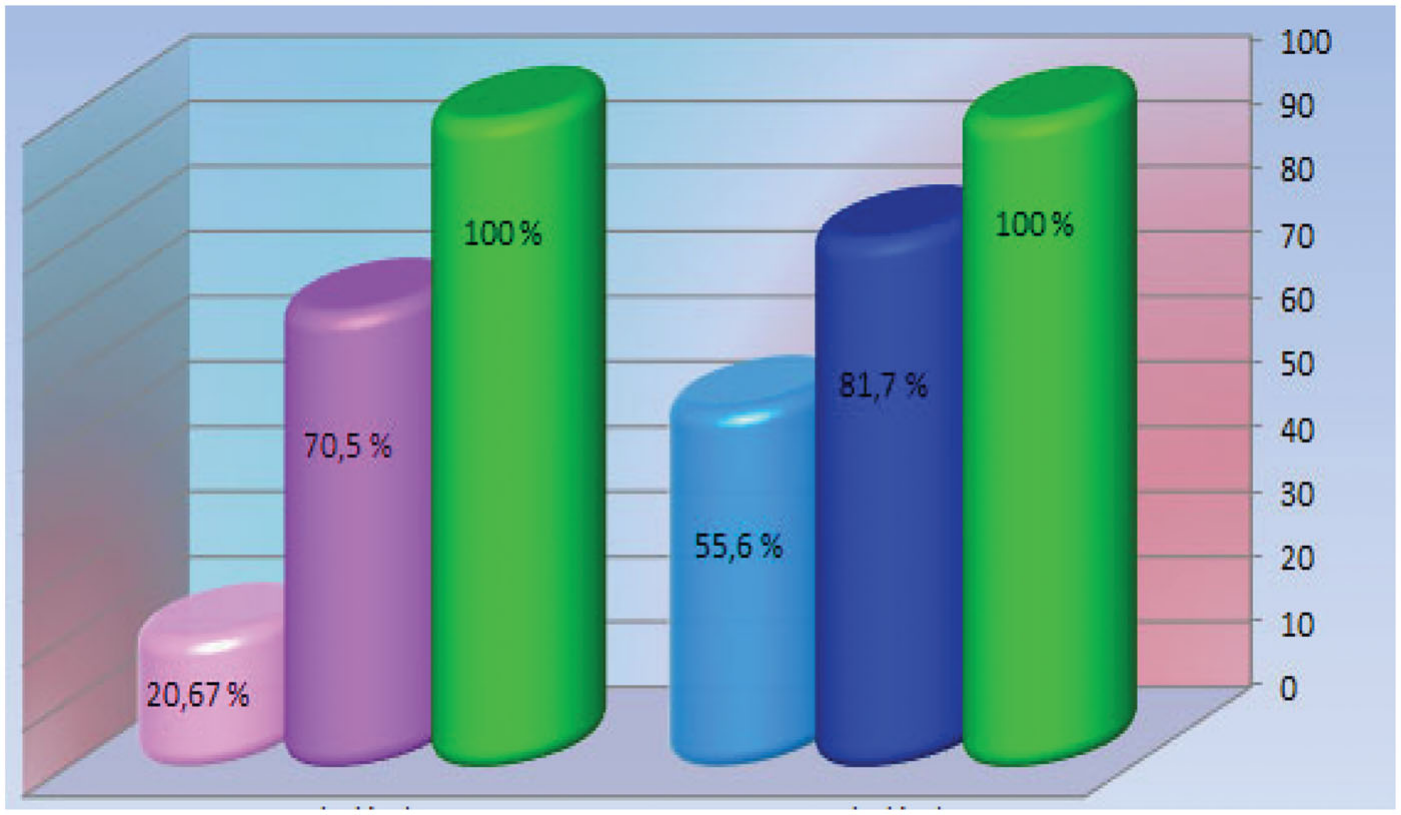
The incidence of hyper-Lp-LPA_2_-emy in relation to the sex of patients in the studied group. 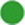 Martor lot. 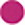 Incidence of increased Lp-PLA2 in females, reported only in females. 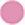 Incidence of Lp-PLA2 increase in females, reported in the entire control group. 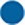 Incidence of male Lp-PLA2 increase, reported only in male cases. 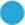 Incidence of male Lp-PLA2 increase, reported in the entire control group.

d. Irrespective of the sex of the patients studied, the incidence of elevated Lp-LPA_2_ serum values calculated on the basis of inpatient determinations compared with the same parameter calculated at discharge on the basis of inpatient dosing shows the predominance of the former, including regarding circulating levels (Table 1, respectively Figure 5).

**Figure 5.**
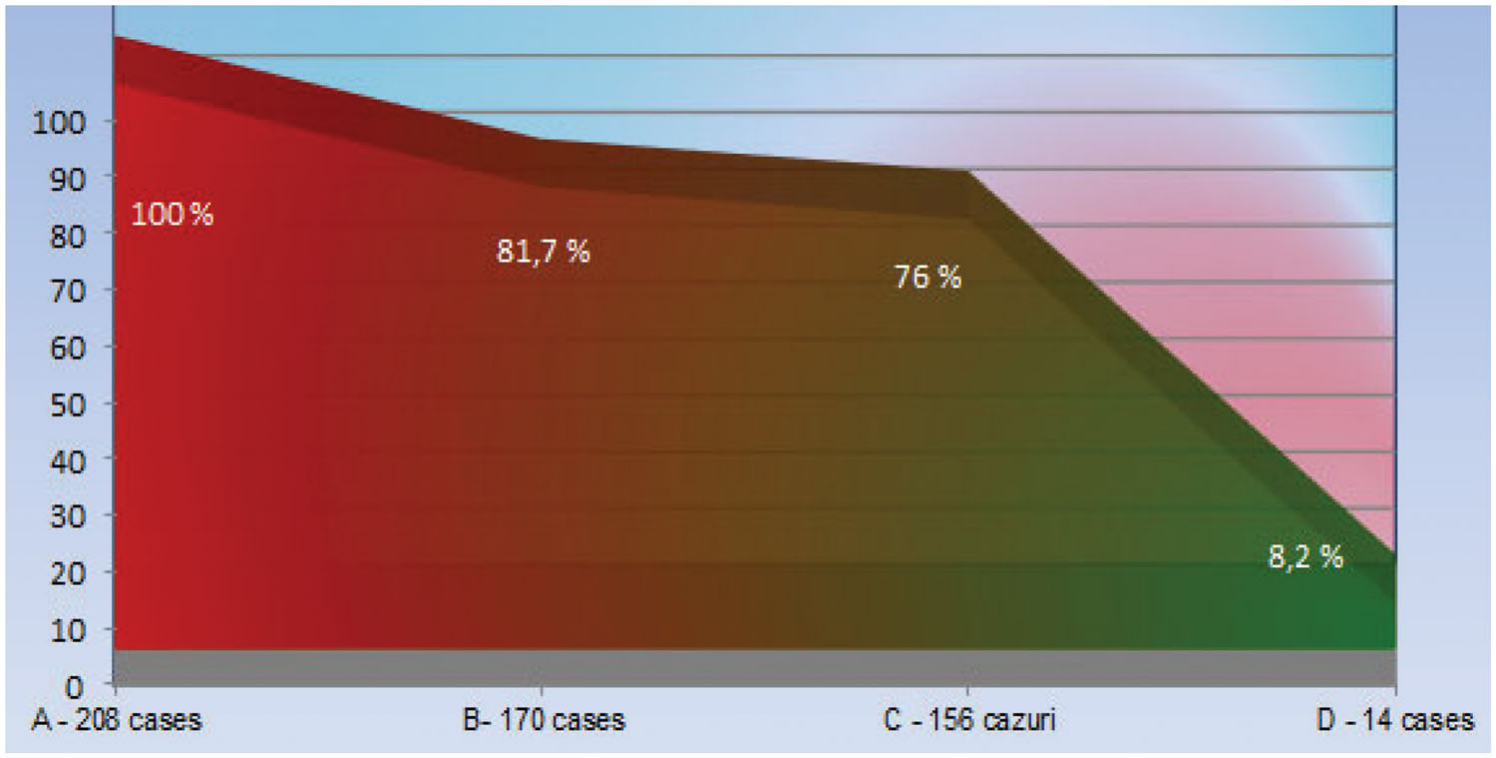
The incidence of medium serum values of Lp-LPA_2_ at the two dosing times and the percentage reduction under the established treatment of the incidence at discharge, compared to that at admission. (A) Number of cases studied (n: 208 = 100%); (B) incidence of hyper-Lp-LPA_2_-emy on admittance, regardless of sex (n: 163 = 78.3%); (C) number of cases with hyper-Lp-LPA_2_-emy on discharge, regardless of sex (n: 156 = 76%); (D) the number of cases in which a decrease in the increased value of the serum level of Lp-PLA_2_, by comparing the incidence at hospital admission with that at discharge (n: 14 = 8.2%).

**Table 1.** Classification of calculation results.

Classification	BMI (kg/m^2^)
Underweight	<18.5
Normal weight	18.51–24.99
Overweight	25.0–29.99
Obese	
Type I	30.0–34.99
Type II	35.0–39.99
Type III	>40

## Discussion

In support of the opportunity of our study, to identify possible diagnostic biomarkers in ischaemic heart disease, come at least two of the current concerns about cardiovascular disease, such as:

a. Their epidemiology, in which atherosclerosis is a major public health disease, generating multiple other cardiovascular diseases, labelled in medical practice as complications from it. Of these, we appreciate that chronic ischaemic heart disease with its multiple forms of manifestation was selected based on sound arguments, if we consider the repercussions on the quality of life of the individual, and the economic cost to the family and society^3^^,^^5^^,^^25–27^.

b. The quasi-permanent concern to identify biomarkers that would allow on the one hand the early evaluation of both atherosclerosis and especially of its various forms of clinical manifestation, in order to optimise the treatment, in order to delay the appearance of its severe forms.

Trying to respond to such a desire for patients already diagnosed with chronic ischaemic heart disease, we investigated possible clinical information that can provide us with medium serum values of Lp-PLA_2_ concentration, calculated based on collection times. For this purpose, we used their comparison with each other and with the one admitted as normal (Table 3).

**Table 3.** Serum parameters used for comparison.

Medium serum concentration/batch regardless of dosing time compared to normal serum value
Medium serum concentration/batch determined at admission versus normal circulating value
Medium serum concentration/batch determined at discharge versus normal blood value
Medium serum concentration/batch determined at admission versus medium serum concentration/batch determined at discharge

In order to interpret the results of the mentioned comparisons, we resorted to determining the value of the p index, admitting the thresholds recorded in the literature as statistically representative^14^.

1. Thus, the medium serum concentration of Lp-PLA_2_ in the component patients of the whole group calculated as a half-sum of the results of the dosages performed at admission and discharge, respectively, was 28.1% higher than the normal circulating value of the marker (Table 1). Statistically, the existing value difference allows us to admit that *the serum value of Lp-PLA_2_ can be considered a biomarker for the diagnosis of chronic ischaemic heart disease*, especially in clinical forms that cannot be certified by the results of other paraclinical examinations performed.

2. The study of the data from Table 1, respectively, Figure 1 highlights that, regardless of gender, the incidence of elevated values of the medium serum concentration calculated for the whole group, regardless of the time of determination of Lp-LPA_2_, is present in a percentage of 78.3, which allows us to propose the parameter *as diagnostic biomarker of chronic ischaemic heart disease*.

3. In support of the proposal that the serum level of Lp-PLA_2_ may be a possible risk biomarker in chronic ischaemic heart disease come the values of the incidence of hyper-Lp-LPA2-emy found at admission and discharge (Table 1 and Figure 5).

4. The study data led to a significant concordance of the increase in the average circulating level of Lp-PLA_2_ with that of its incidence, especially for men, which is why we postulate not only that the enzyme can be supported as a biomarker in chronic ischaemic heart disease, but also that *it manifests specificity in relation to the patient’s sex*.

5. Related to the latter finding, in relation to the sex of the patients studied, the data showed that the incidence of hyper-Lp-PLA_2_-emy present in men is 2.1 times higher than that corresponding to women (Figure 4). In patients of the same sex, the incidence of increased hyper-LPA_2_-emy is 7 out of 10 women, and for men 8 out of 10, which means a value of the risk of developing serum growth of Lp-LPA_2_ substantially equal, i.e. unrelated to sex. We explain this by the composition of the study group, which in the sub-batch of women included mostly cases at climax and post-menopause. Through such a selection, the protective actions induced by oestrogen hormones were minimised.

6. We note that the conclusions of the study specified in points 3 and 4, according to which in men with chronic ischaemic heart disease there is a significant increase in Lp-PLA_2_ serum concentration, but which is not associated with an increase in incidence, i.e. sex-dependent, are not specified in the specialised literature. If we take into account the fact that in inter-current medical practice the interpretation of serum phospholipase concentrations is made by comparison with values admitted as normal, but which do not vary with the person’s sex, we appreciate that through this study we generated a new possible research objective in the field, to identify whether or not there is a sex-related difference in enzyme serum values, even in healthy individuals.

At the same time, we recognise as a limitation of the study undertaken by us that for a real support of the highlighted specificity it is necessary to carry out research on quantitatively significant groups consisting of people considered healthy, respectively with chronic ischaemic heart disease.

7. The lipid-lowering and coronary dilator therapy instituted for the patients in the group led, at discharge, to the reduction of the medium circulating concentration of Lp-PLA_2_ compared to that dosed at admission with an approximate rate of 10%. We interpret the value of this quota as expressing the beneficial effect of the established therapy. Although it is a small reduction in the value of the average circulating concentration of Lp-PLA2, we appreciate that it correlates and expresses the results of the short therapy of the disease of only 2 weeks. Indirectly, we appreciate that the percentage reduction rate may be a motivation for a significant study in terms of duration of therapy and case series, in order to argue the possible proposal of Lp-PLA_2_ serum level also as a marker to assess the evolution of myocardial ischaemia under treatment, thus constituting a *possible risk biomarker in this disease*.

## Conclusions

The conclusions of our study on patients with only chronic ischaemic heart disease lead to a high percentage of increased circulating levels of Lp-PLA_2_, but also its incidence, which is why we postulate that the enzyme can be supported as a biomarker in chronic ischaemic heart disease. The particular way of “behaviour” of the enzyme in the sub-batch of men opens the new direction of research to determine whether the serum value of Lp-PLA_2_ depends on the sex of the patient, even in people considered healthy or if the serum behaviour of the enzyme has a specificity of sex only for chronic ischaemic heart disease.

At the same time, the study data allows us to make the assumption that the circulating level of Lp-PLA_2_ can be evaluated as a possible biomarker of risk and assessment of the response to a certain type of lipid-lowering therapy instituted in patients with chronic ischaemic heart disease.
